# Supplementary material for: Network medicine links SARS-CoV-2/COVID-19 infection to brain microvascular injury and neuroinflammation in dementia-like cognitive impairment
Source: Alzheimers Res Ther. 2021 Jun 9;13:110. doi: 10.1186/s13195-021-00850-3 (PMC8189279; doi:10.1186/s13195-021-00850-3)
Supplement: Supplementary file 1 — Additional file 1: Figure S1. Functional enrichment analysis and largest connected component of the six CRISPR-Cas9-based SARS-CoV-2 host factor datasets. Figure S2. Network proximity results using different numbers of top genes from the CRISPR-Cas9-based SARS-CoV-2 host factor datasets. Figure S3. Single-cell level expression of AD blood markers in the PBMC samples of COVID-19 patients. Figure S4. Expression spectrum of the SARS-CoV-2 entry factors in the entorhinal cortex from Alzheimer’s disease patients and controls. Figure S5. Expression spectrum of the SARS-CoV-2 entry factors in individuals with different APOE genotypes. Figure S6. Expression of the key SARS-CoV-2 entry factors in different tissues. Figure S7. Expression of the key SARS-CoV-2 entry factors in different brain regions. Figure S8. Cumulative degree distribution of 964 innate immune genes, 14267 brain expressed genes, and 3383 brain specific genes. [file 13195_2021_850_MOESM1_ESM.pdf]

SUPPORTING INFORMATION

Network medicine links SARS-CoV-2/COVID-19 infection to brain microvascular injury and neuroinflammation in dementia-like cognitive impairment

Zhou Y et al., 2021

*Correspondence to: Feixiong Cheng, PhD

Lerner Research Institute, Cleveland Clinic

Tel: 216-444-7654; Fax: 216-636-0009

Email: chengf@ccf.org

Table of Contents

Supplementary Results

Supplementary Figures

Fig. S1. Functional enrichment analysis and largest connected component of the six CRISPR-Cas9-based SARS-CoV-2 host factor datasets.

Fig. S2. Network proximity results using different numbers of top genes from the CRISPR-Cas9-based SARS-CoV-2 host factor datasets.

Fig. S3. Single-cell level expression of AD blood markers in the PBMC samples of COVID-19 patients.

Fig. S4. Expression spectrum of the SARS-CoV-2 entry factors in the entorhinal cortex from Alzheimer's disease patients and controls.

Fig. S5. Expression spectrum of the SARS-CoV-2 entry factors in individuals with different *APOE* genotypes.

Fig. S6. Expression of the key SARS-CoV-2 entry factors in different tissues.

Fig. S7. Expression of the key SARS-CoV-2 entry factors in different brain regions.

Fig. S8. Cumulative degree distribution of 964 innate immune genes, 14267 brain expressed genes, and 3383 brain specific genes.

Supplementary Tables

Table S1. SARS-CoV-2 host factor datasets. (.xlsx)

Table S2. Neurological diseases-associated genes/proteins. (.xlsx)

Table S3. Alzheimer's disease markers and their expressions. (.xlsx)

Table S4. Transcriptomic datasets used in this study.

Table S5. Raw data and network analysis results of the nodes in Fig. 2b. (.xlsx)

Table S6. Differentially expressed genes in brain endothelial cells vs. other cell types. (.xlsx)

Table S7. Differentially expressed genes in brain endothelial cells by comparing *APOE* genotype E3/E3 and E4/E4 in Alzheimer's disease patients. (.xlsx)

Table S8. Differentially expressed genes in Alzheimer's disease patients vs. normal controls for each cell type. (.xlsx)

Supplementary Results

We compiled ten SARS-CoV-2 and other HCoVs host factor profiles, including six datasets from CRISPR-Cas9 assays (CRISPR_A549-H, CRISPR_A549-L, CRISPR_HuH7-229E, CRISPR_HuH7-OC43, CRISPR_HuH7-SARS2, and CRISPR_VeroE6), and four datasets for virus-human PPIs (SARS2-PPI, SARS1-PPI, MERS-PPI, and HCoV-PPI) (see Methods). The six CRISPR-Cas9-based datasets adopted genome-scale CRISPR loss-of-function screening methods in the SARS-CoV-2 infected cell lines (as indicated in the dataset name) to identify host factors required for the infection.

As we hypothesized that the SARS-CoV-2 host factors form a subnetwork within the comprehensive human protein interactome, we first computed the largest connected components (LCC) of the CRISPR-Cas9-based datasets. LCC quantifies the number of genes/proteins in the largest subnetwork formed by a dataset. We found that three of these datasets, including CRISPR_A549-H, CRISPR_A549-L, and CRISPR_HuH7-229E, consistently show significantly large LCC (**Table S1**), when we used top-50, -100, and -150 genes. Top-100 revealed the highest number of significant LCCs for the SARS-CoV-2 datasets (CRISPR_A549-H $p = 0.007$, CRISPR_A549-L $p < 0.001$, CRISPR_VeroE6 $p = 0.037$, permutation test, **Table S1**, **Fig. S1**). Therefore, we selected top-100 genes from these datasets for downstream analyses. These results suggest that these datasets form disease modules in the human protein interactome and offer opportunities for network-based discoveries.

Next, we performed functional enrichment analyses for these datasets (**Fig. S1**). We identified several common pathways and GO terms that are enriched in more than

three datasets, including autophagy, lysosome, vesicle-mediated transport, endosomal transport, intracellular pH reduction, macromolecule catabolic process, regulation of lysosomal lumen pH, cytosolic transport, and selective autophagy. These datasets also have different functional enrichment. For example, CRISPR_VeroE6 is enriched in functions related to cell cycle, cell growth, and chromatin remodeling, and CRISPR_HuH7-SARS2 is enriched in heparan sulfate biosynthetic functions. These results suggest that the SARS-CoV-2 host factors participate in various essential cellular functions. In addition, these datasets contain complementary information of the cellular states of the SARS-CoV-2 infection and host response.

Table S4. Transcriptomic datasets used in this study.

GEO ID	Type	Organism	Sample / Brain region	Groups	Cell types
GSE147528	single-nuclei RNA-seq	Homo sapiens	superior frontal gyrus and entorhinal cortex	10 males with varying stages of Alzheimer's disease (AD)	astrocytes, excitatory neurons, inhibitory neurons, and microglia
GSE157827	single-nuclei RNA-seq	Homo sapiens	prefrontal cortex	12 AD patients and 9 normal controls	astrocytes, endothelial cells, excitatory neurons, inhibitory neurons, microglia, and oligodendrocytes
GSE138852	single-nuclei RNA-seq	Homo sapiens	entorhinal cortex	AD (n = 6) and healthy controls (n = 6)	astrocytes, endothelial cells, neurons, microglia, oligodendrocytes, and oligodendrocyte progenitor cells
GSE157103	bulk RNA-seq	Homo sapiens	peripheral blood mononuclear cell (PBMC)	66 intensive care unit (ICU) patients (COVID-19 patients n = 50 vs. non-COVID-19 patients n = 16), 59 non-ICU patients (COVID-19 patients n = 49 vs. non-COVID-19 patients n = 10), and all 125 patients	N/A
GSE149689	single-cell RNA-seq	Homo sapiens	PBMC	6 samples from severe COVID-19 patients, 4 samples from mild COVID-19 patients, and 4 samples from healthy controls	IgG^- B cells, IgG^+ B cells, $CD4^+$ T cell effector memory (EM)-like cells, $CD4^+$ T cell non-EM-like cells, $CD8^+$ T cell EM-like cells, $CD8^+$ T cell non-EM-like cells, dendritic cells, monocytes, intermediate monocytes, nonclassical monocytes, natural killer cells, platelets, and red blood cells
GSE163005	single-cell RNA-seq	Homo sapiens	Cerebrospinal fluid	8 COVID-19 patients, 9 multiple sclerosis patients, 9 idiopathic intracranial hypertension patients, and 5 viral encephalitis patients	T cells, dendritic cells, and monocytes

Fig. S1

CRISPR_A549-H

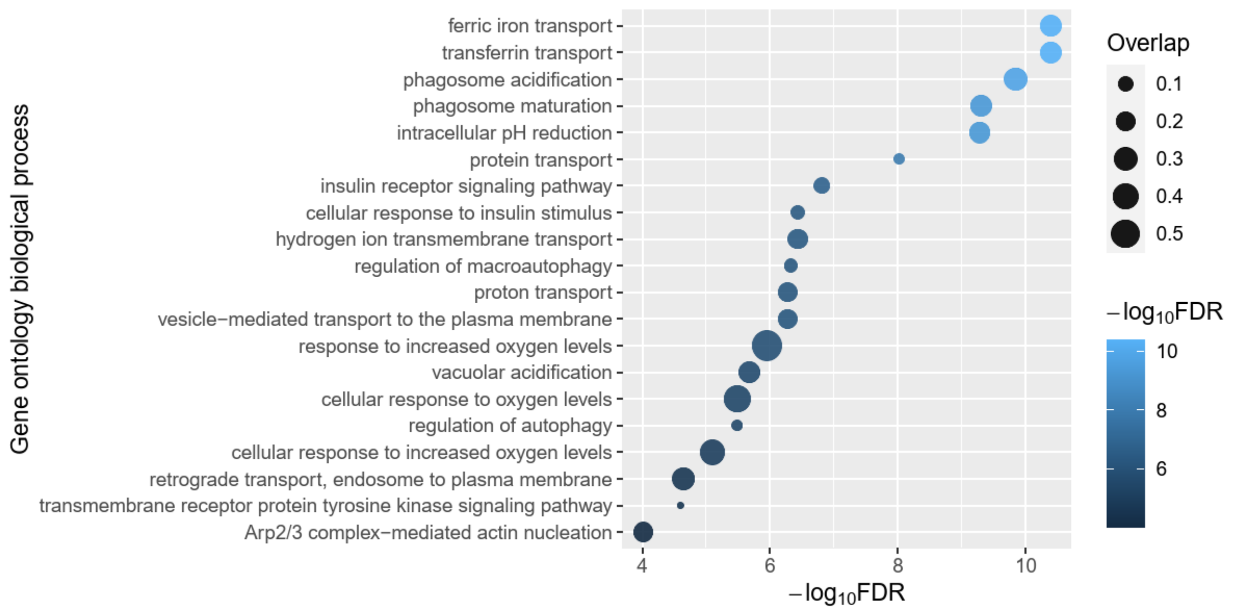

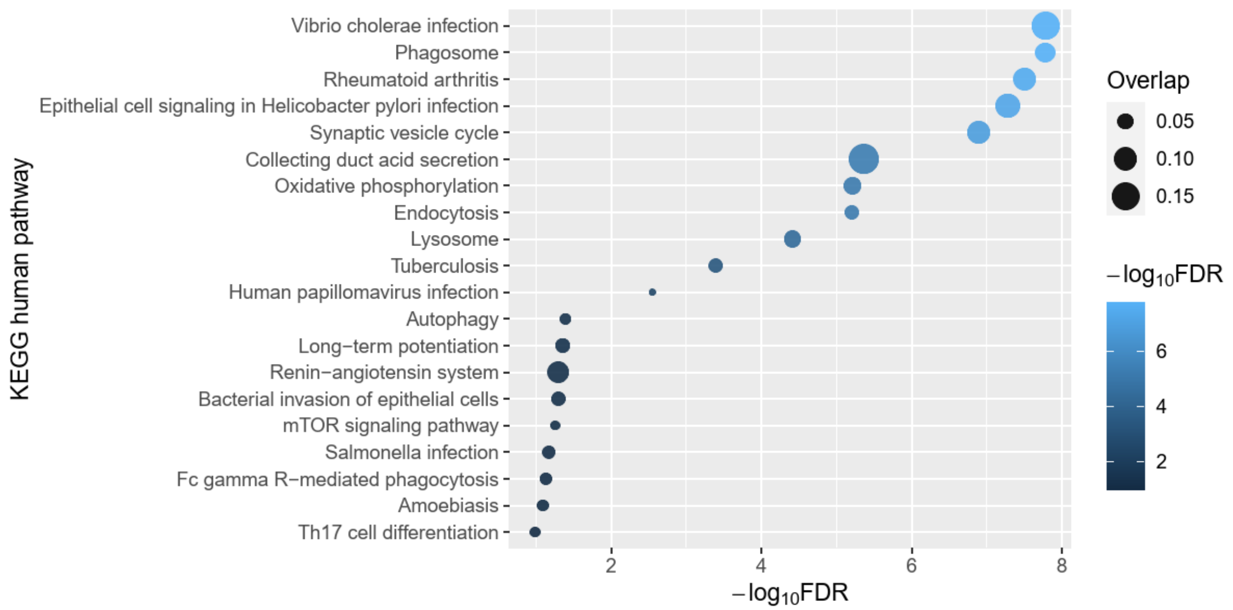

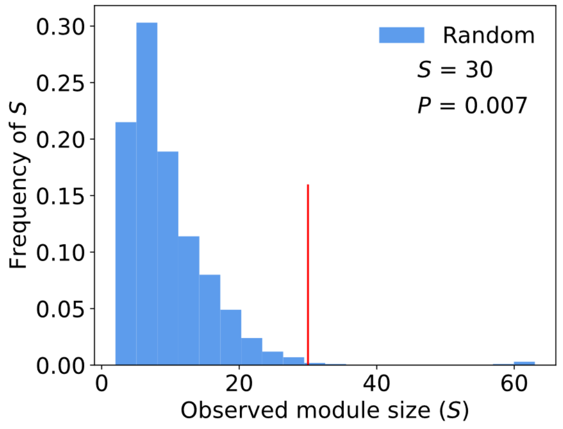

Fig. S1. Functional enrichment analysis and largest connected component of the six CRISPR-Cas9-based SARS-CoV-2 host factor datasets. Top 100 genes from each dataset were used for the analyses.

Fig. S1 continued

CRISPR_A549-L

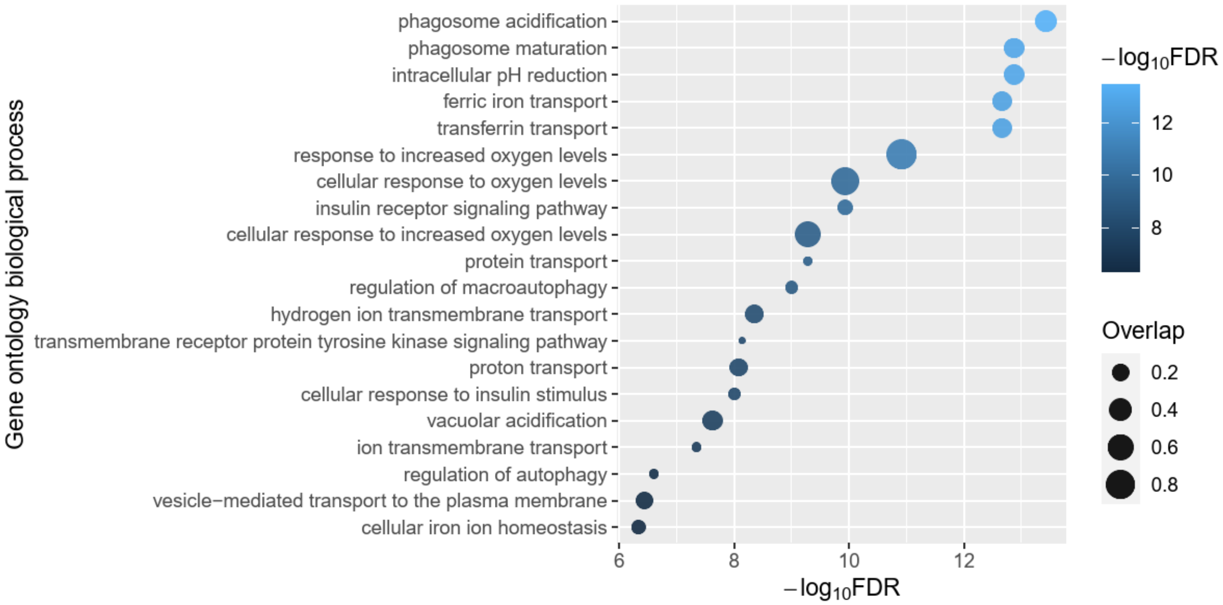

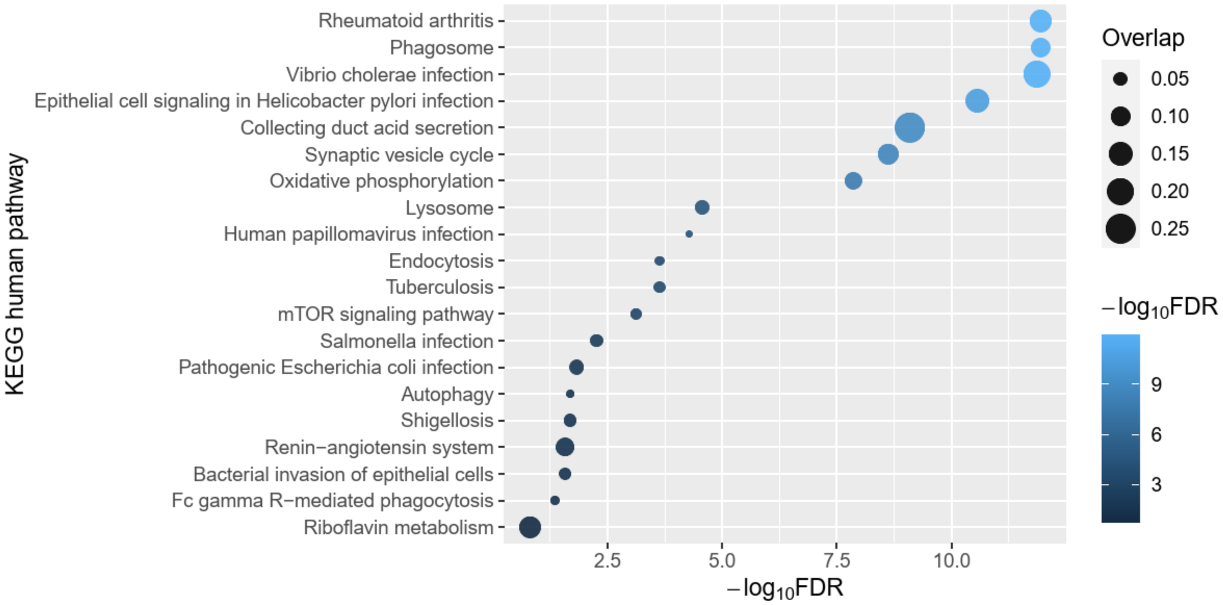

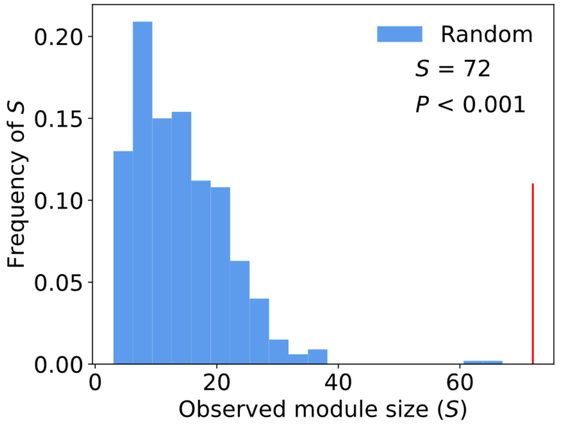

Fig. S1 continued

CRISPR_HuH7-229E

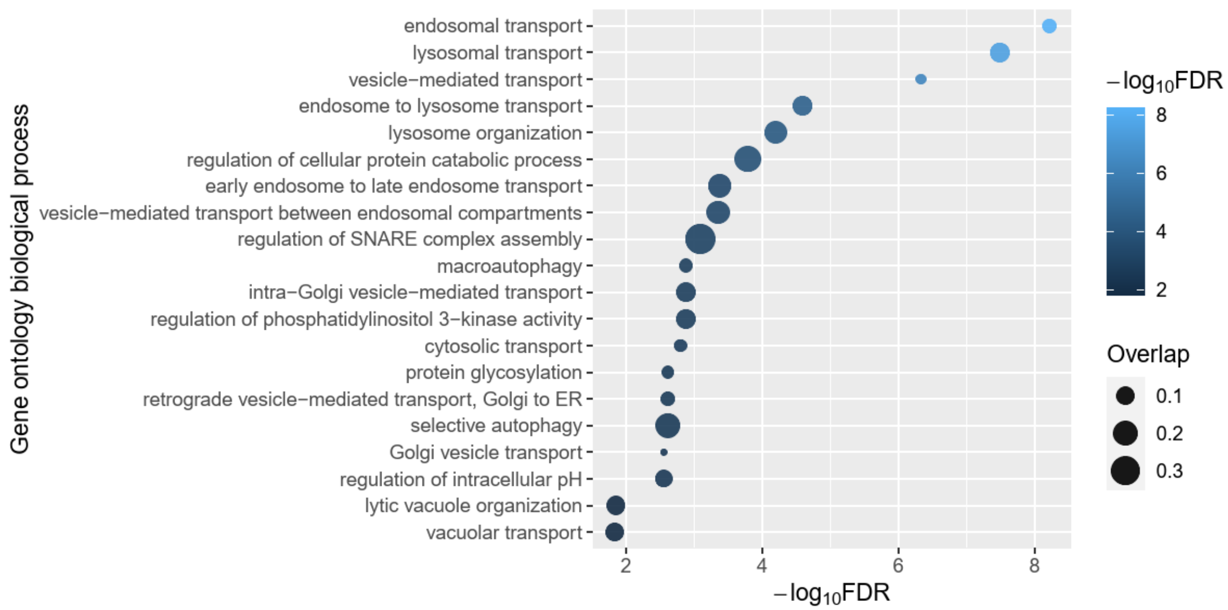

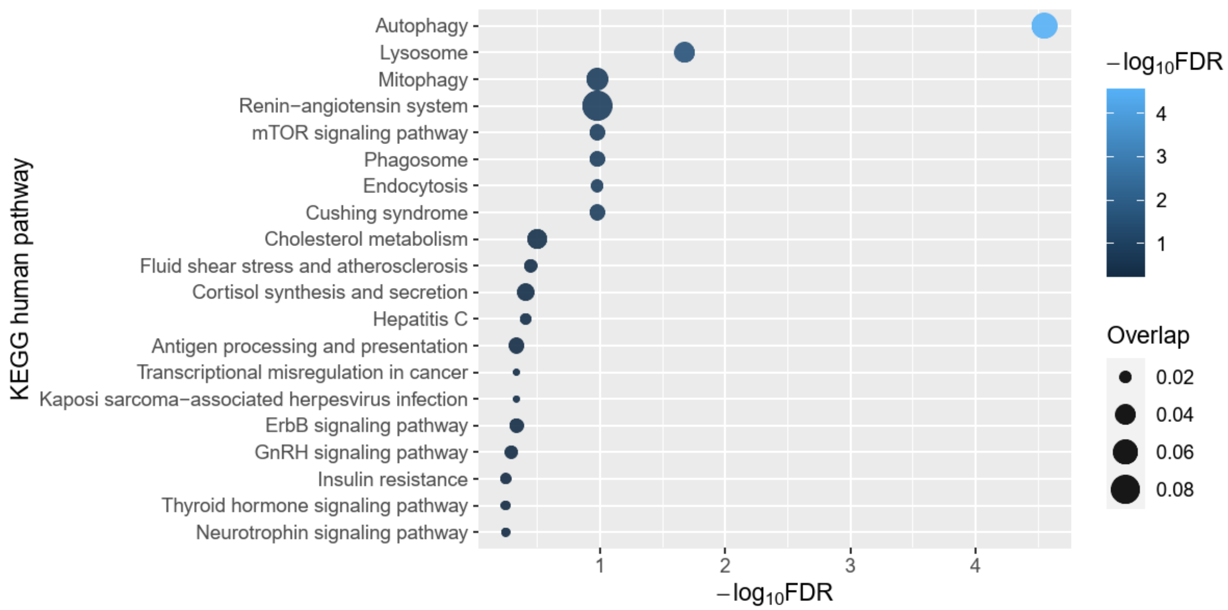

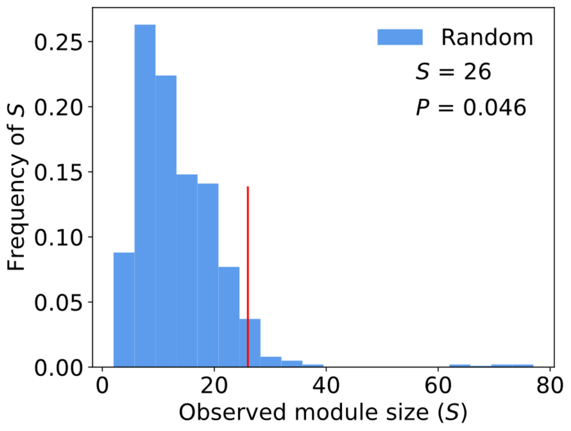

Fig. S1 continued

CRISPR_HuH7-OC43

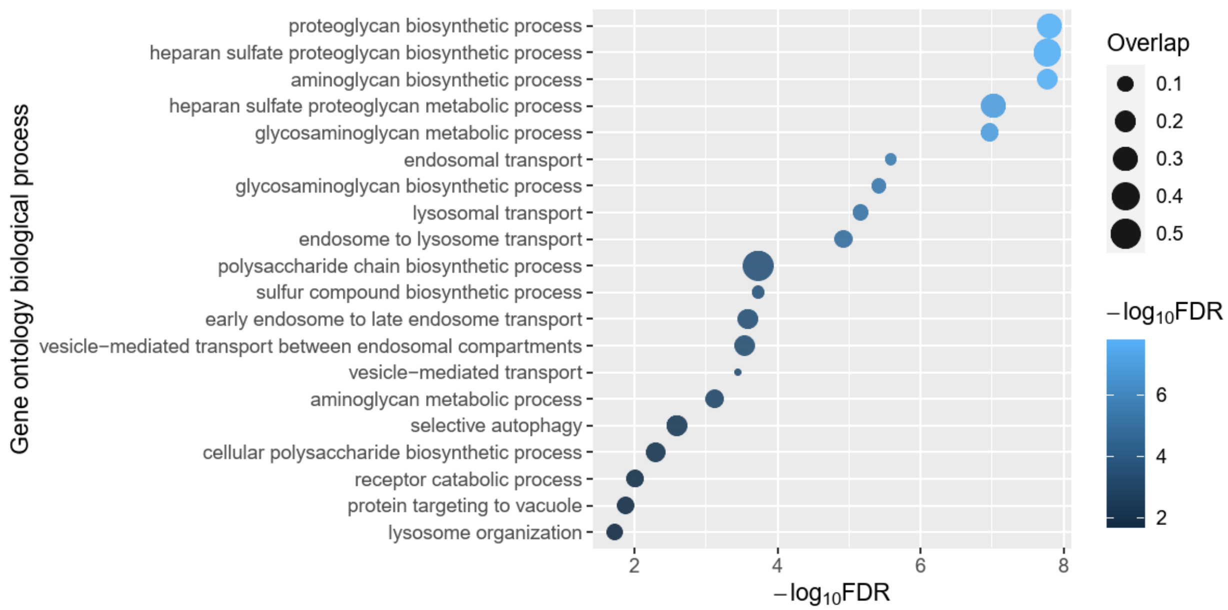

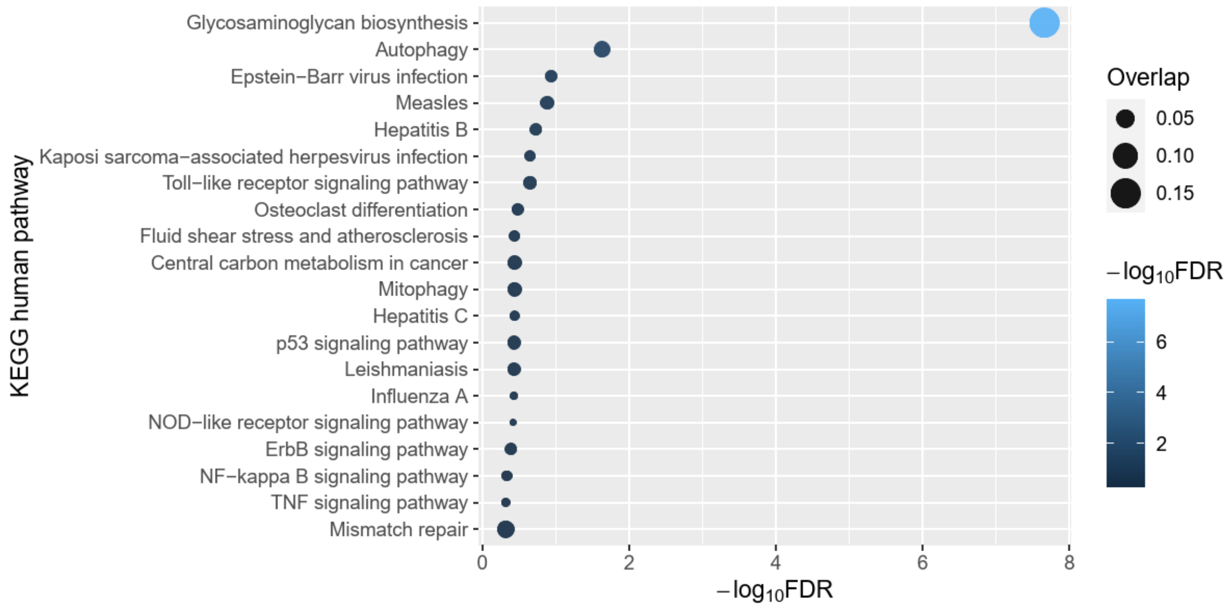

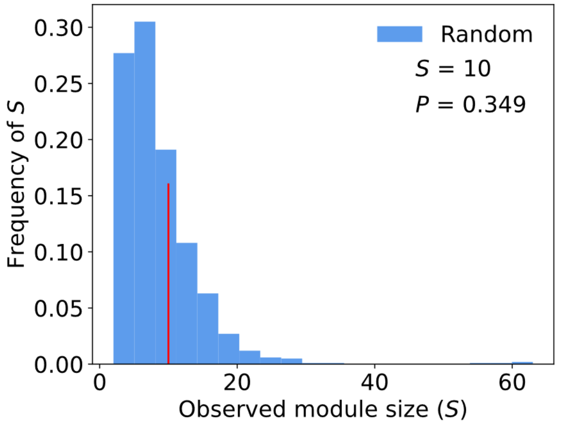

Fig. S1 continued

CRISPR_HuH7-SARS2

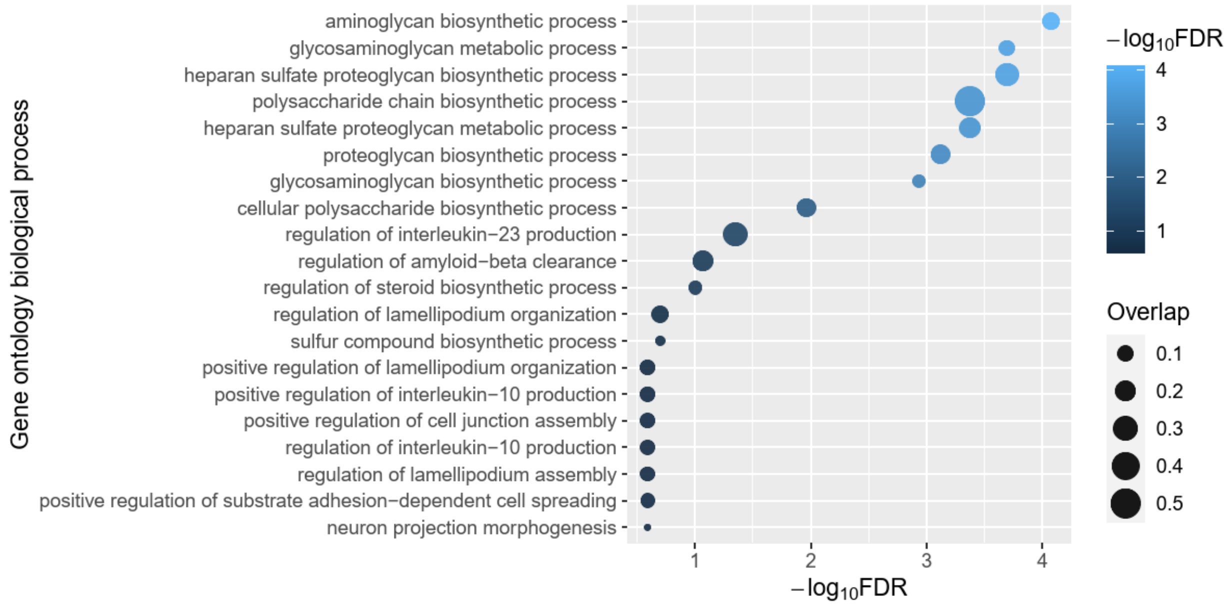

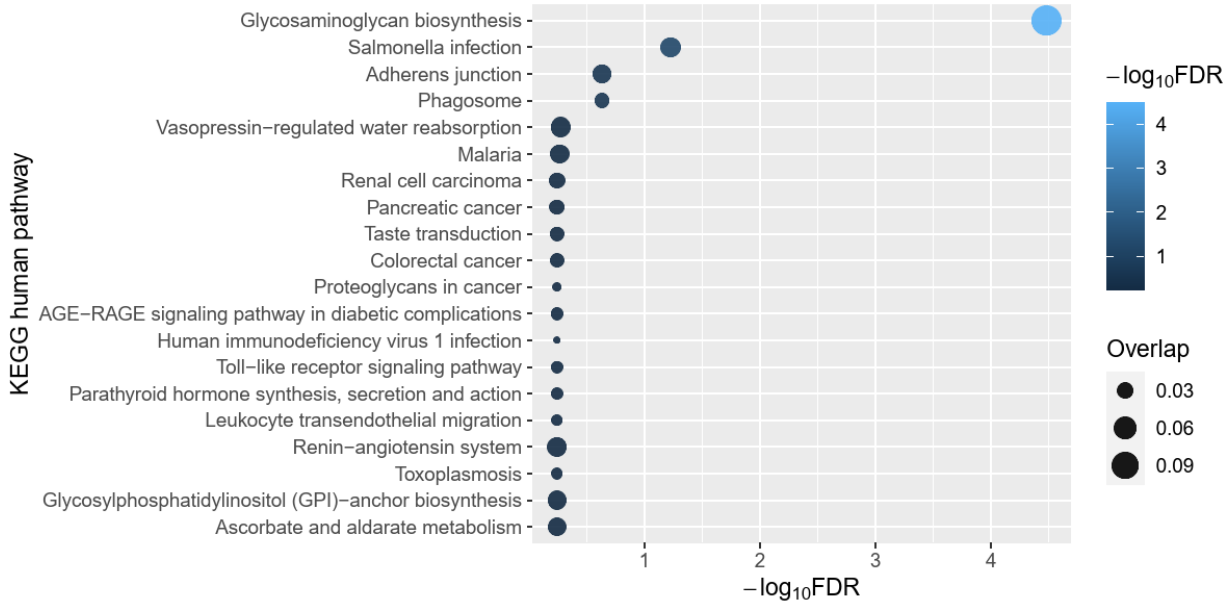

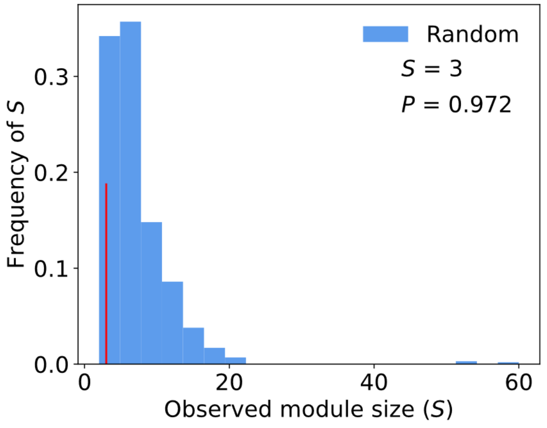

Fig. S1 continued

CRISPR_VeroE6

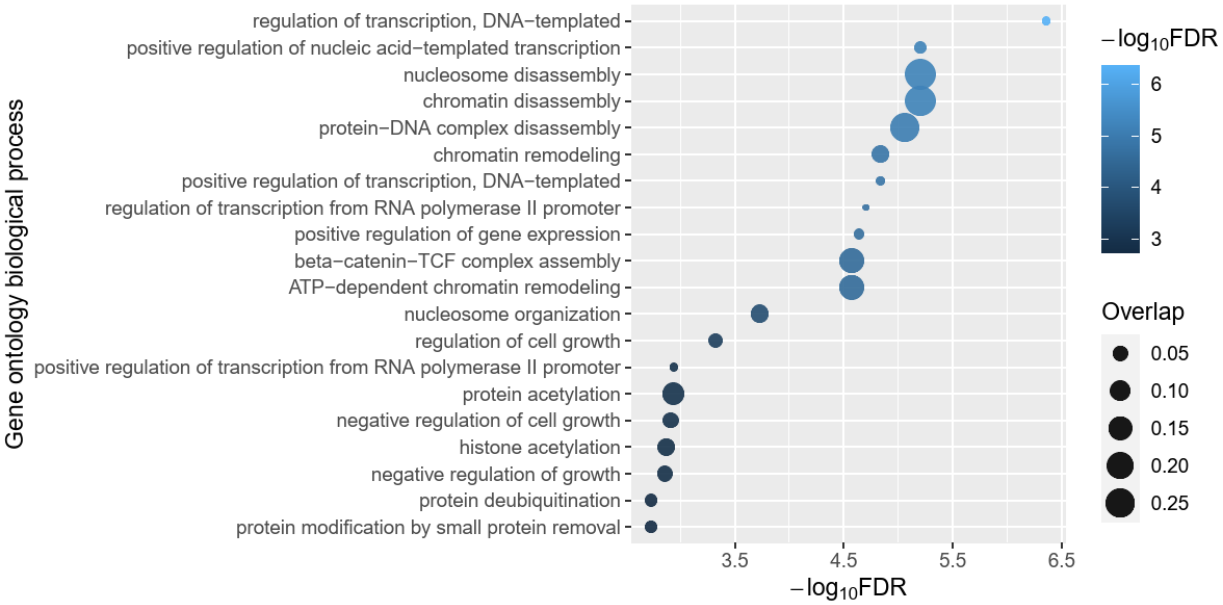

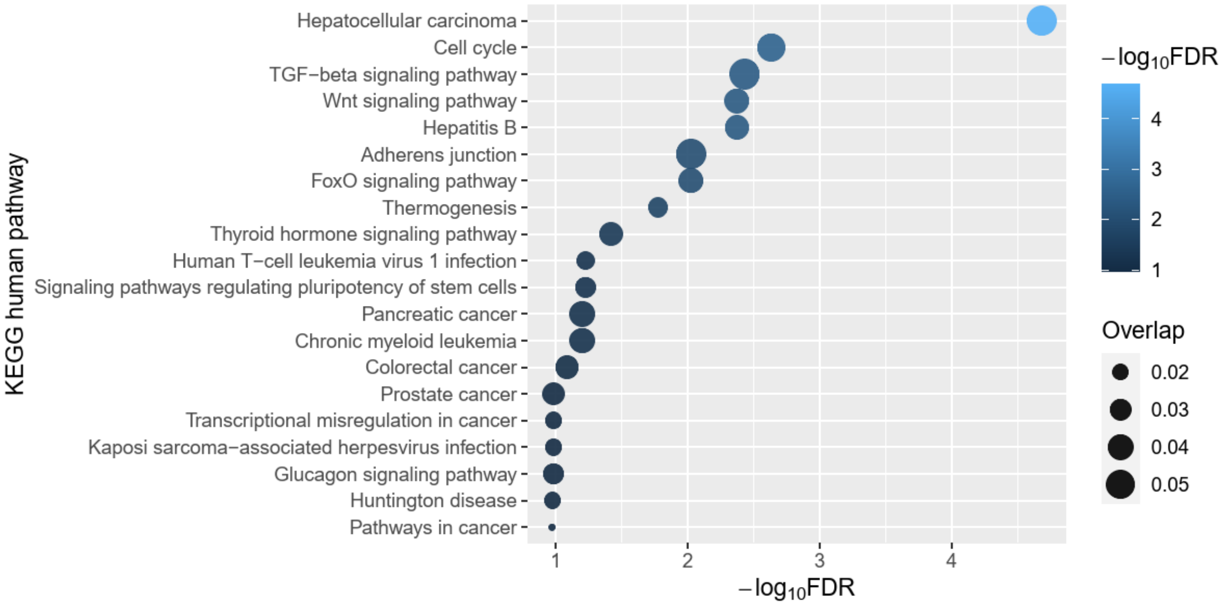

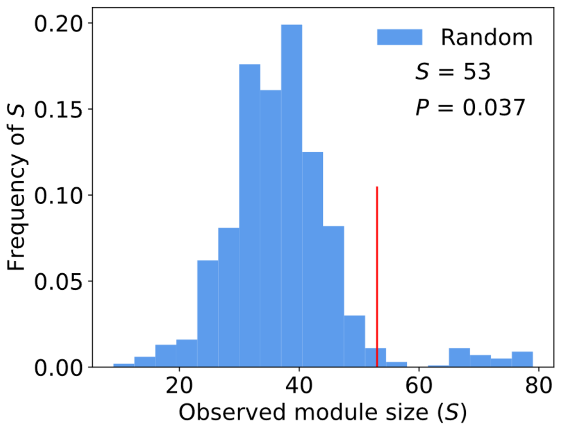

Fig. S2

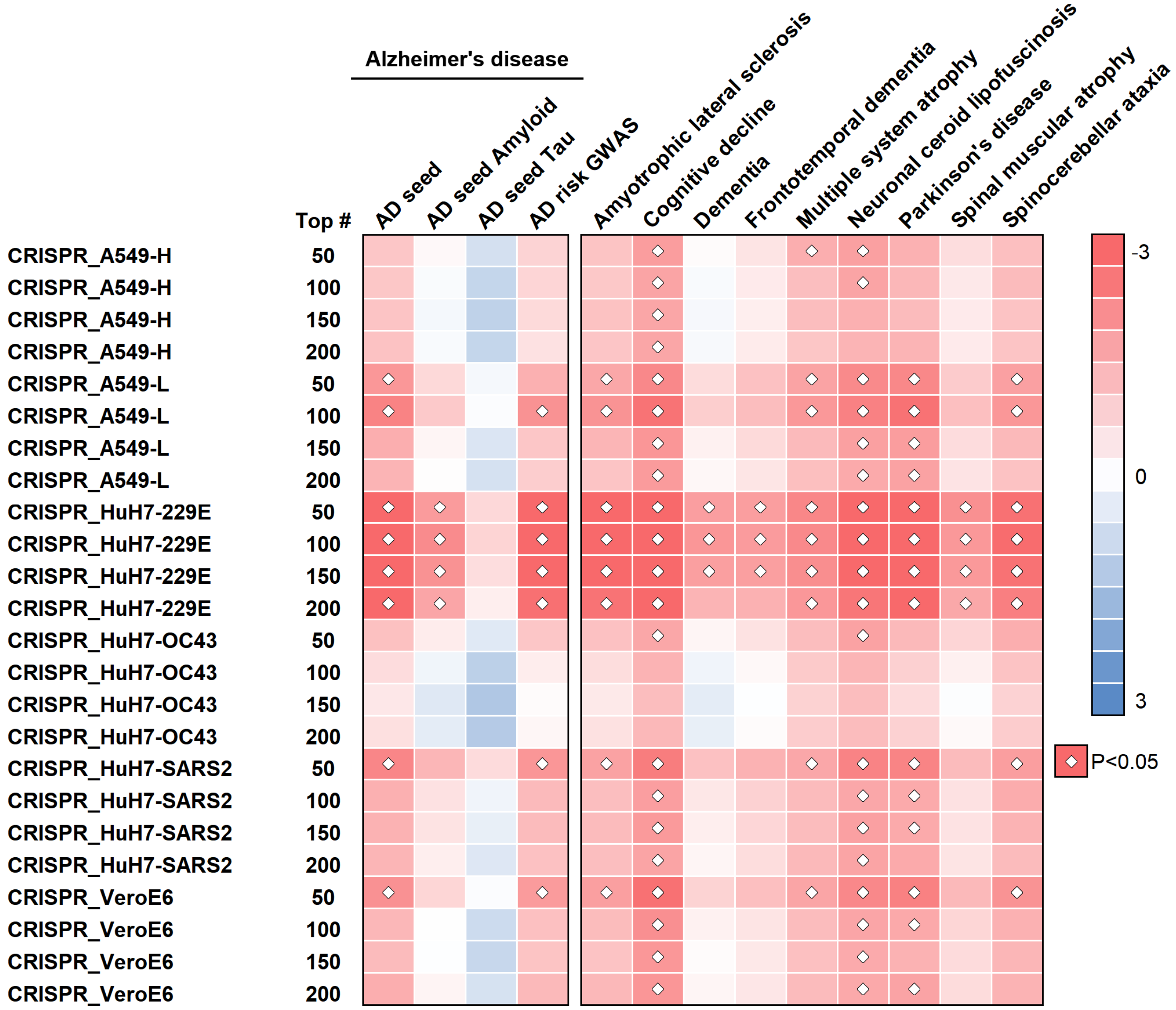

Fig. S2. Network proximity results using different numbers of top genes from the CRISPR-Cas9-based SARS-CoV-2 host factor datasets. Heatmap shows the proximities of the CRISPR-Cas9-based SARS-CoV-2 host factor datasets and 10 neurological diseases using different numbers of top genes (i.e., top-50, -100, -150, and -200) from the CRISPR-Cas9 assay.

Fig. S3

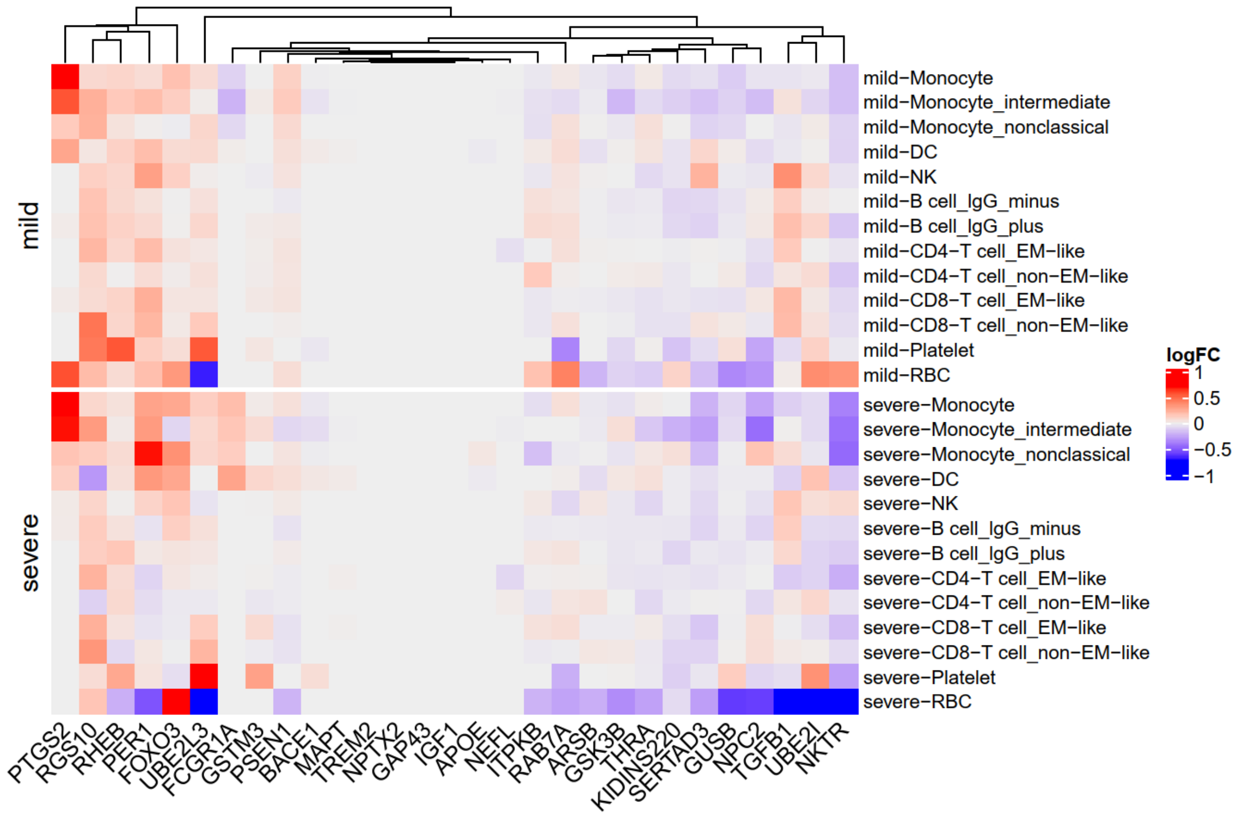

Fig. S3. Single-cell level expression of AD blood markers in the PBMC samples of COVID-19 patients. Heatmap shows the expression change in mild / severe COVID-19 patients versus healthy controls. Data source: GSE149689.

Fig. S4

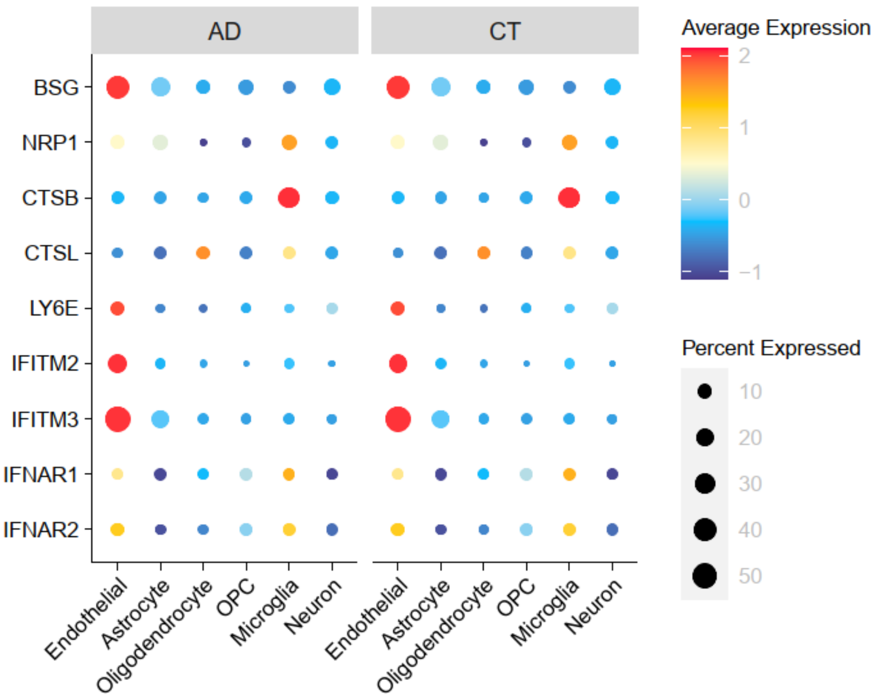

Fig. S4. Expression spectrum of the SARS-CoV-2 entry factors in the entorhinal cortex from Alzheimer's disease patients and controls. AD, Alzheimer's disease patients. CT, controls. OPC, oligodendrocyte progenitor cell. Data source: GSE138852.

Fig. S5

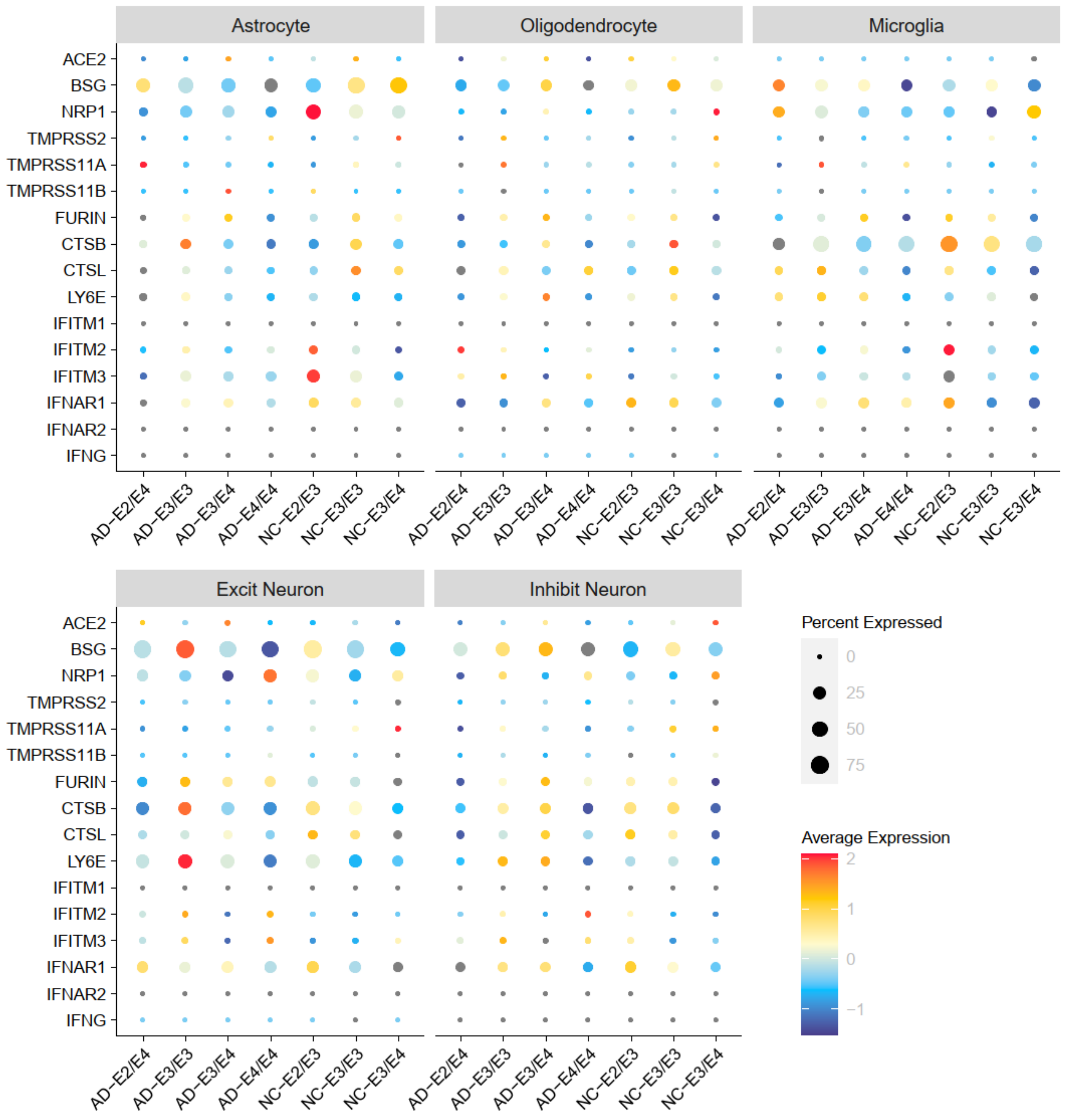

Fig. S5. Expression spectrum of the SARS-CoV-2 entry factors in individuals with different *APOE* genotypes. AD, Alzheimer's disease patients. NC, normal controls. Data source: GSE157827.

Fig. S6

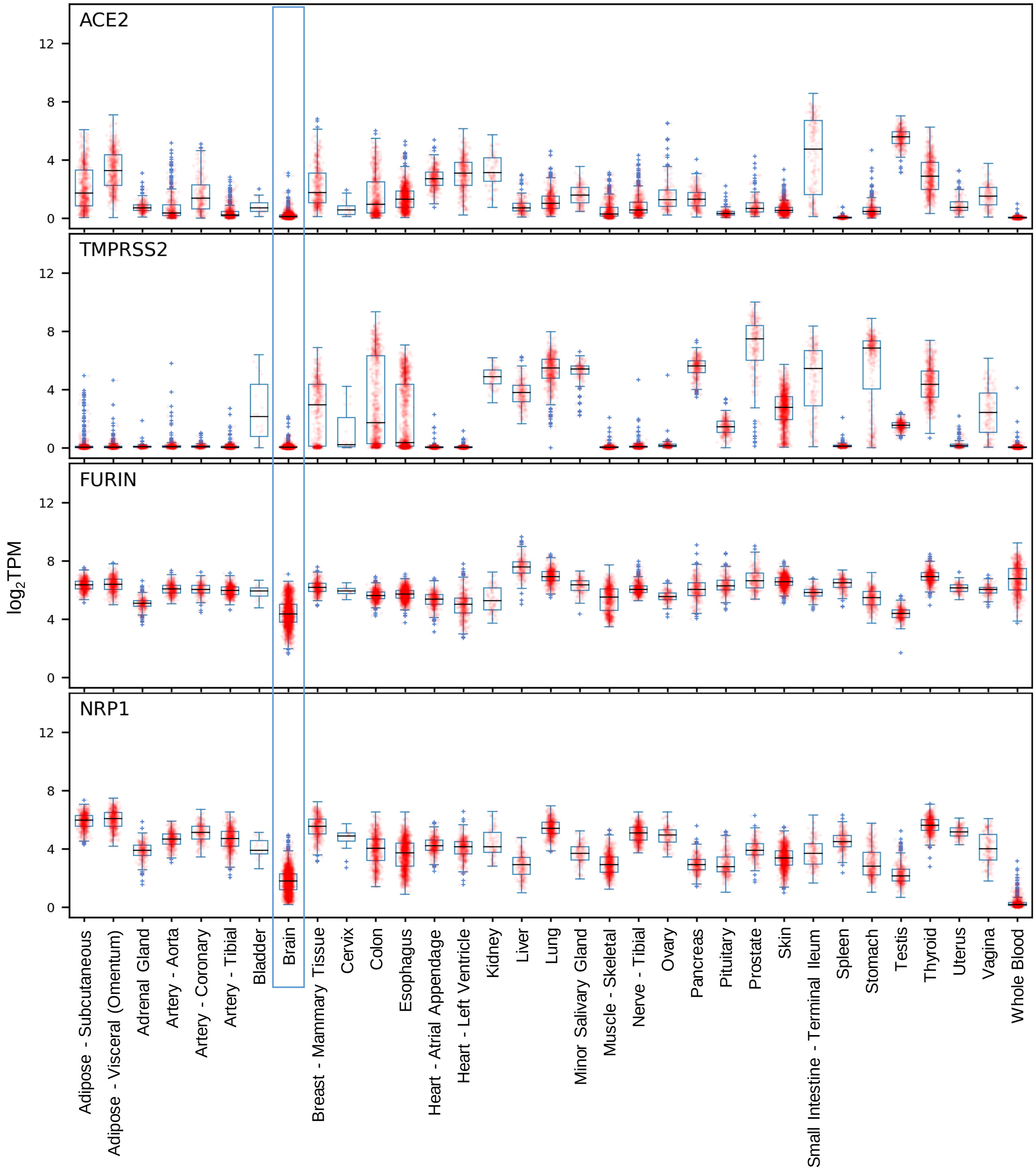

Fig. S6. Expression of the key SARS-CoV-2 entry factors in different tissues. Data source: GTEx v8.

Fig. S7

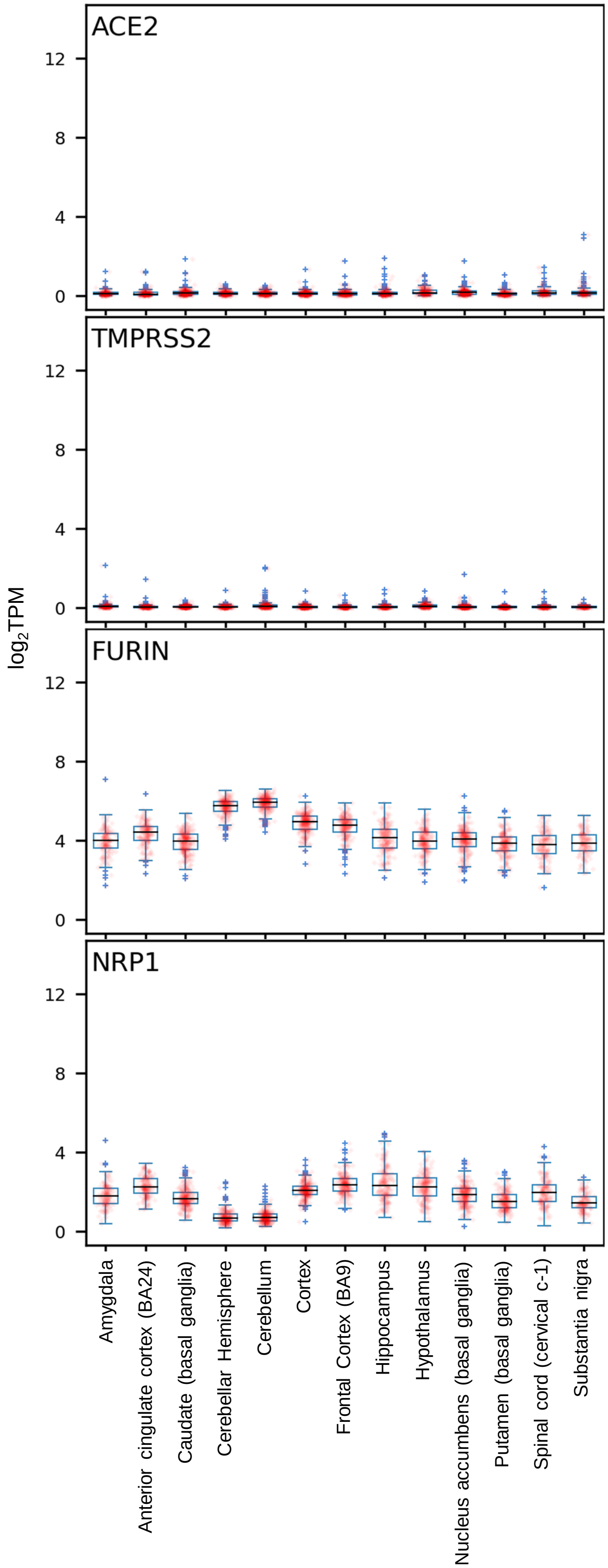

Fig. S7. Expression of the key SARS-CoV-2 entry factors in different brain regions. Data source: GTEx v8.

Fig. S8

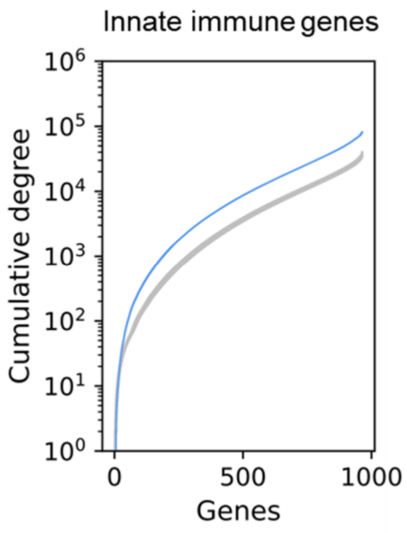

Fig. S8. Cumulative degree distribution of 964 innate immune genes, 14267 brain expressed genes, and 3383 brain specific genes. Blue - degree of these gene sets; grey – background generated using permutation test of 100 repeats with the entire interactome. Genes that were in the interactome were used for sampling. Innate immune genes were extracted from InnateDB. Brain expressed and brain specific genes were generated based on the GTEx data using the method described in the manuscript (genes with count per million ≥ 0.5 in over 90% samples were considered expressed; genes with positive normalized average expression were considered specific).
