# Supplementary material for: Network medicine links SARS-CoV-2/COVID-19 infection to brain microvascular injury and neuroinflammation in dementia-like cognitive impairment
Source: Alzheimers Res Ther. 2021 Jun 9;13:110. doi: 10.1186/s13195-021-00850-3 (PMC8189279; doi:10.1186/s13195-021-00850-3)
Supplement: Supplementary file 2 [file 13195_2021_850_MOESM5_ESM.docx]

Table S4. Transcriptomic datasets used in this study.

GEO ID	Type	Organism	Sample / Brain region	Groups	Cell types
GSE147528	single-nuclei RNA-seq	Homo sapiens	superior frontal gyrus and entorhinal cortex	10 males with varying stages of Alzheimer's disease (AD)	astrocytes, excitatory neurons, inhibitory neurons, and microglia
GSE157827	single-nuclei RNA-seq	Homo sapiens	prefrontal cortex	12 AD patients and 9 normal controls	astrocytes, endothelial cells, excitatory neurons, inhibitory neurons, microglia, and oligodendrocytes
GSE138852	single-nuclei RNA-seq	Homo sapiens	entorhinal cortex	AD (n = 6) and healthy controls (n = 6)	astrocytes, endothelial cells, neurons, microglia, oligodendrocytes, and oligodendrocyte progenitor cells
GSE157103	bulk RNA-seq	Homo sapiens	peripheral blood mononuclear cell (PBMC)	66 intensive care unit (ICU) patients (COVID-19 patients n = 50 vs. non-COVID-19 patients n = 16), 59 non-ICU patients (COVID-19 patients n = 49 vs. non-COVID-19 patients n = 10), and all 125 patients	N/A
GSE149689	single-cell RNA-seq	Homo sapiens	PBMC	6 samples from severe COVID-19 patients, 4 samples from mild COVID-19 patients, and 4 samples from healthy controls	IgG^- B cells, IgG^+ B cells, $CD4^+$ T cell effector memory (EM)-like cells, $CD4^+$ T cell non-EM-like cells, $CD8^+$ T cell EM-like cells, $CD8^+$ T cell non-EM-like cells, dendritic cells, monocytes, intermediate monocytes, nonclassical monocytes, natural killer cells, platelets, and red blood cells
GSE163005	single-cell RNA-seq	Homo sapiens	Cerebrospinal fluid	8 COVID-19 patients, 9 multiple sclerosis patients, 9 idiopathic intracranial hypertension patients, and 5 viral encephalitis patients	T cells, dendritic cells, and monocytes
